# A widely-used eddy covariance gap-filling method creates systematic bias in carbon balance estimates

**DOI:** 10.1038/s41598-023-28827-2

**Published:** 2023-01-31

**Authors:** Henriikka Vekuri, Juha-Pekka Tuovinen, Liisa Kulmala, Dario Papale, Pasi Kolari, Mika Aurela, Tuomas Laurila, Jari Liski, Annalea Lohila

**Affiliations:** 1 Finnish Meteorological Institute, 00101 Helsinki, Finland; 2 DIBAF University of Tuscia, 01100 Viterbo, Italy; 3 Euro-Mediterranean Center on Climate Change CMCC IAFES, 01100 Viterbo, Italy; 4 Institute for Atmospheric and Earth System Research, Physics, University of Helsinki, 00014 Helsinki, Finland

**Keywords:** Atmospheric science, Carbon cycle

## Abstract

Climate change mitigation requires, besides reductions in greenhouse gas emissions, actions to increase carbon sinks in terrestrial ecosystems. A key measurement method for quantifying such sinks and calibrating models is the eddy covariance technique, but it requires imputation, or gap-filling, of missing data for determination of annual carbon balances of ecosystems. Previous comparisons of gap-filling methods have concluded that commonly used methods, such as marginal distribution sampling (MDS), do not have a significant impact on the carbon balance estimate. By analyzing an extensive, global data set, we show that MDS causes significant carbon balance errors for northern (latitude $>60^\circ$) sites. MDS systematically overestimates the carbon dioxide (CO$_2$) emissions of carbon sources and underestimates the CO$_2$ sequestration of carbon sinks. We also reveal reasons for these biases and show how a machine learning method called extreme gradient boosting or a modified implementation of MDS can be used to substantially reduce the northern site bias.

## Introduction

Climate change is one of the most severe challenges faced by humankind. In addition to limiting greenhouse gas emissions from fossil fuels and land use, it is necessary to find effective ways of sequestering carbon (C), most notably carbon dioxide (CO$_2$), already present in the atmosphere. Natural climate solutions, such as climate-smart agriculture, afforestation, reforestation and peatland restoration, are considered the most feasible means for this^1,2^. To make these solutions credible for climate policy and carbon markets, reliable verification of carbon sequestration is necessary^3^. The verification also involves the micrometeorological eddy covariance (EC) technique, a key method to directly measure the CO$_2$ fluxes between ecosystems and the atmosphere^4^. The popularity of this method is manifest in the FLUXNET network, which has had over 900 EC sites worldwide registered over the years^5^. In principle, EC provides continuous data on the short-term net ecosystem exchange (NEE) of CO$_2$ with the atmosphere, which can be integrated temporally to determine the related carbon balance of an ecosystem. Even though EC measurements can be run continuously, in practice there are gaps in the collected data, for example due to technical failures and, most importantly, due to the need for filtering of the data collected in atmospheric conditions compromising the validity of the EC technique. For example, in the global FLUXNET2015 data set, with 1532 site-years of data^6^, on average 68% of the half-hourly CO$_2$ fluxes are missing^7^. Even if the site-years that have gaps longer than two months are excluded, the mean data coverage is 40%. Only 50 site-years have a coverage higher than 60% and only 5 site-years a coverage higher than 70%.

Various methods have been used to impute, or gap-fill, missing data, with methods ranging from simple linear interpolation and mean diurnal variation to more complex methods such as artificial neural networks (ANN). In a comparison of 15 CO$_2$ flux gap-filling methods, it was concluded that the effect of gap-filling is modest on the annual C balance and that the accuracy of the best-performing methods, which proved to be nonlinear regression, look-up table, marginal distribution sampling (MDS), a semi-parametric model and ANN, is already reaching the noise limit of measurements^8^. However, this comparison only included forest sites from a $20^\circ$ latitudinal range. Other comparisons have missed the gap-filling methods most commonly used nowadays^9^, namely MDS and machine learning-based approaches, or focused on long gaps^7,10,11^. Despite these shortcomings, different machine learning methods and especially MDS have become the standard methods for gap-filling EC data. Notably, MDS is used for gap-filling the standardized open-access NEE data provided by FLUXNET^6^ and the European research infrastructure Integrated Carbon Observation System (ICOS). MDS is also implemented within the free gap-filling tool REddyProc^12^ and as part of Tovi$^{TM}$, a commercial software for EC data post-processing^13^. However, knowledge on the performance of different gap-filling methods, especially MDS, is lacking for data from northern high-latitude (latitude $>60^\circ$) sites. In northern ecosystems, growing seasons are short and the amount of solar radiation, a key environmental driver in CO$_2$ exchange, is distributed very unevenly throughout the year. Therefore, the amount of potentially available nighttime data during the short northern growing season is low even before filtering for data quality.

Here, we investigated the performance of two methods to gap-fill CO$_2$ flux time series with the aim to reveal if the gap-filling-induced uncertainties limit our abilities to verify carbon sequestration estimates in northern latitudes. The gap-filling methods considered were MDS and a machine learning method called eXtreme gradient boosting (XGBoost). XGBoost was chosen from a wide range of machine learning methods because decision tree-based methods have performed well in previous studies on gap-filling CO$_2$ and methane flux data^7,10,14^. First, we compared the performance of MDS and XGBoost on the global FLUXNET2015 data set including annual time series of half-hourly data from the northern hemisphere that had at least a 20% temporal CO$_2$ flux data coverage. Next, we focused on the special case of northern ecosystems by investigating gap-filling performance for ten northern sites. We addressed a basic gap-filling task involving a maximum of three-day data gaps and the use of only readily available environmental drivers: air temperature (T$_{air}$), incoming shortwave radiation (SWR) and atmospheric vapour pressure deficit (VPD). More specifically, we wanted to know if the gap-filling methods (1) result in similar C balance estimates and (2) perform equally well in northern ecosystems as elsewhere. We did this by inserting artificial gaps into real and synthetic data sets, gap-filling the artificial gaps, and evaluating the performance of both methods. We adopted the mean flux bias as the main performance metric, as it directly translates to the accuracy of an annual balance. Another key metric that was used was the balance error, which is the sum of flux biases over missing data. This was calculated using synthetic data.

## Results

### Skewed radiation distribution causes a positive bias at northern sites when using MDS

**Figure 1:**
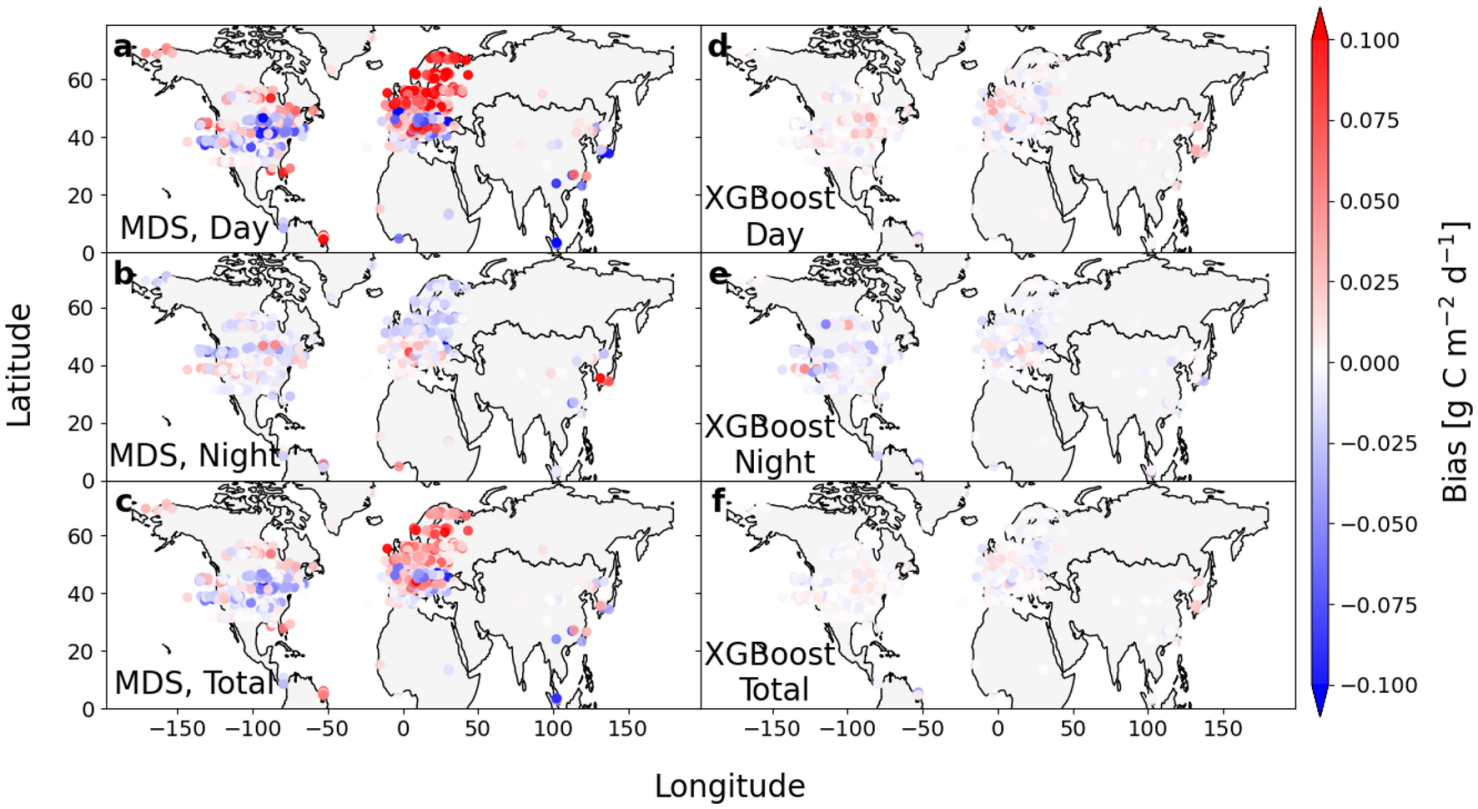
MDS causes a clear positive bias in the gap-filled NEE at northern latitudes. (**a**,**b**) Daytime, (**c**,**d**) nighttime and (**e**,**f**) total bias in the CO$_2$ flux data gap-filled using marginal distribution sampling (MDS) and extreme gradient boosting (XGBoost). The data cover 882 site-years from 141 sites from the global FLUXNET2015 data set. Outputs of each method were compared to measured data labelled as artificial gaps. The total, daytime and nighttime mean flux bias is plotted for each site-year.

In this work, the micrometeorological sign convention is adopted, which means that a positive CO$_2$ flux denotes a flux from the ecosystem to the atmosphere and a negative flux indicates uptake from the atmosphere into the ecosystem. Gap-filling of artificial gaps inserted into 882 site-years of data in the FLUXNET2015 data set revealed that with MDS there was a clear positive bias in the gap-filled fluxes during daytime (SWR $\ge$ 20 W m$^{-2}$) in high latitudes ($>50^\circ$N, N = 105) (Fig. 1a; for statistical tests, see Supplementary Table S1). During nighttime (SWR < 20 W m$^{-2}$), there was a negative, but much smaller bias (Fig. 1b) leading to a positive total flux bias (Fig. 1c). A positive flux bias indicates that either emission was overestimated or uptake was underestimated, while a negative bias indicates the opposite. When XGBoost was used for gap-filling, some positive and negative flux biases were observed (Fig. 1d,e), but the magnitude of these biases was small compared to the daytime bias of MDS. The total flux bias with XGBoost was insignificant or very small at all latitudes (Fig. 1f and Supplementary Table S1).

**Figure 2:**
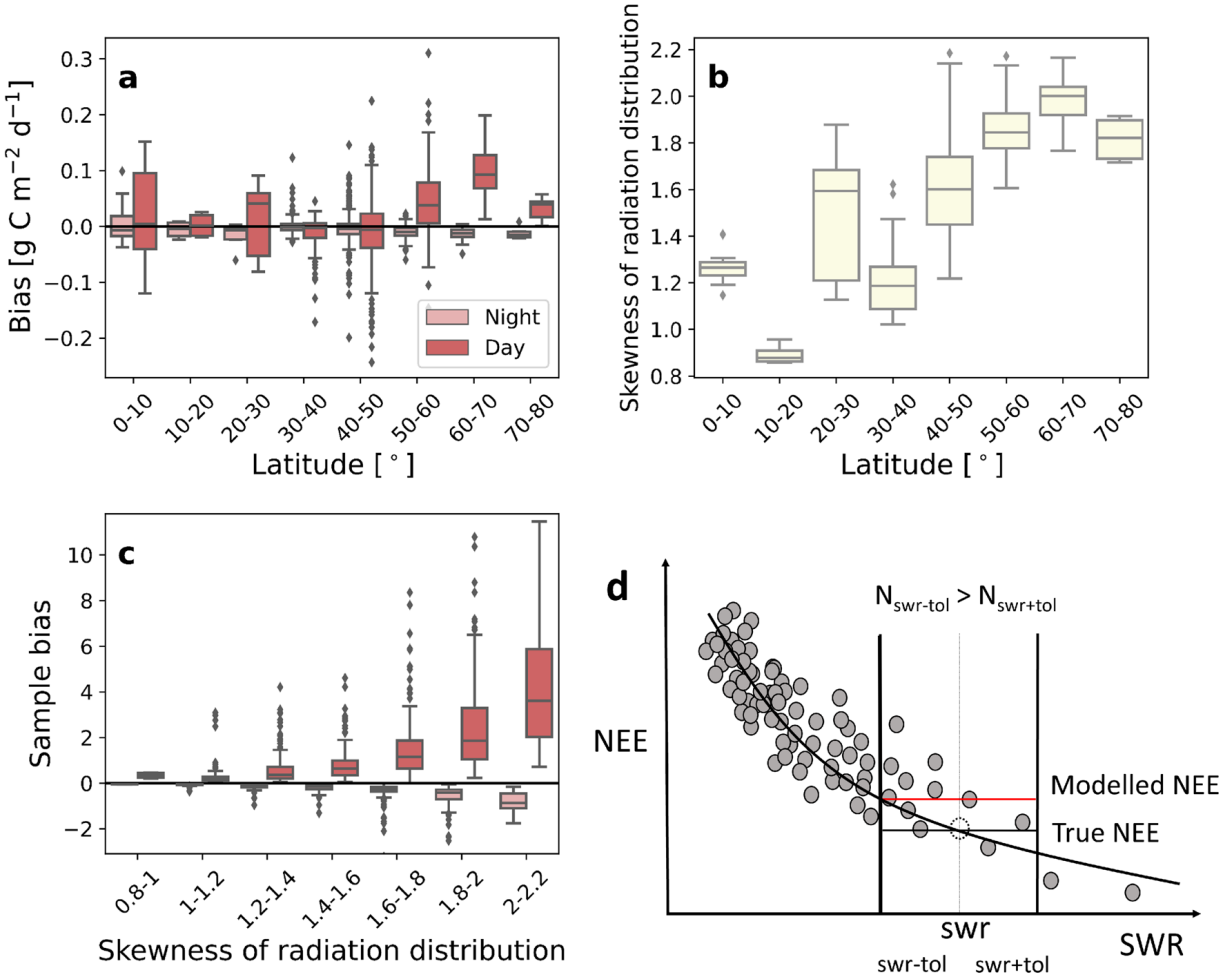
The radiation distribution is skewed at northern latitudes which results in a sample bias and a positive flux bias. (**a**) Daytime and nighttime CO$_2$ flux bias of MDS at different latitudes, (**b**) skewness of the distribution of incoming shortwave radiation at different latitudes, (**c**) sample bias during daytime and nighttime at different skewness values of the radiation distribution and (**d**) a schematic figure illustrating how the positive bias is produced. The black curve shows an example of the true dependence of NEE on SWR (a short period of daytime NEE during the growing season). Sample bias (**c**) indicates how many more data points are sampled with lower than higher radiation values compared to the measured value when selecting the NEE data that are averaged to impute a missing observation. If the number of measurements (N) within the tolerance interval below measured swr (swr-tol...swr) is higher than the number of measurements within the tolerance interval above it (swr...swr+tol), there is a positive sample bias, and the modelled NEE at certain swr is likely to be overestimated. The sample size ranged from 2 to 507 with a mean of 29. The data cover 882 site-years. All boxplots illustrate the median (solid line), quartiles (box) and rest of the distribution, excluding outliers, which are plotted individually. Outliers were defined as points that were outside 1.5 times the interquartile range. Sample biases over 12 (N = 6) and under − 3 (N = 4) are not shown for clarity.

The MDS method is based on the covariation between NEE and meteorological variables and primarily gap-fills a missing NEE observation by the mean of the available NEE values measured under similar conditions. The similarity of meteorological conditions is determined based on sampling tolerances, or maximum accepted deviations from the actual conditions, specified for each predictor variable (for details see Methods). The positive daytime bias at the northern latitudes (Fig. 2a) resulted from a very skewed radiation distribution (Fig. 2b and Supplementary Fig. S1), causing more data to be sampled from the lower range of the radiation distribution (Fig. 2c). An underestimated radiation level corresponds to underestimated photosynthetic uptake (negative flux component) and thus to overestimated NEE (Fig. 2d). Examples using measured data are shown in Supplementary Fig. 2a,b. The imbalance of daytime and nighttime flux bias was obvious at latitudes $50^\circ$–$70^\circ$, affecting 133 site-years of data which corresponds to 15% of the site-years in the selected subset of the FLUXNET2015 data set. At latitudes $30^\circ$–$50^\circ$ there was also a substantial number of site-years with a negative flux bias, indicating that the biases at those latitudes were caused by other reasons. Because the positive daytime flux bias with MDS was especially evident at latitudes $60^\circ$–$70^\circ$, we investigated more thoroughly data from ten sites located within this zone (Table 1). These data were acquired from the ICOS Warm Winter 2020 EC flux product, since there were more site-years with good data coverage available for the northern latitudes^15^. The ICOS data product is fully compatible with the FLUXNET2015 data set.

### Gap-filling of synthetic data reveals C balance errors with MDS

To assess what implications the detected bias has on the annually accumulated CO$_2$ fluxes, i.e. the estimates of annual C balances, we generated synthetic full time series corresponding to the CO$_2$ fluxes observed at the ten northern sites (Supplementary Figs. S3, S4 and Supplementary Table S2). We then introduced realistic, both in length and timing, artificial gaps (30%, 50% and 70% of data) into the synthetic data sets and compared the C balances derived from the gap-filled NEE time series to the true balances. This was done for two reasons. In the first part of the study, we assessed the flux bias based on the available measurement time series that were compromised by data gaps. However, most gaps fall into nighttime, meaning that the majority of the data used to assess the bias were daytime fluxes. Secondly, we wanted to know what is the combined effect of realistic gap lengths, timing and biases, which depend on the time of the day, on estimating the C balance.

**Figure 3:**
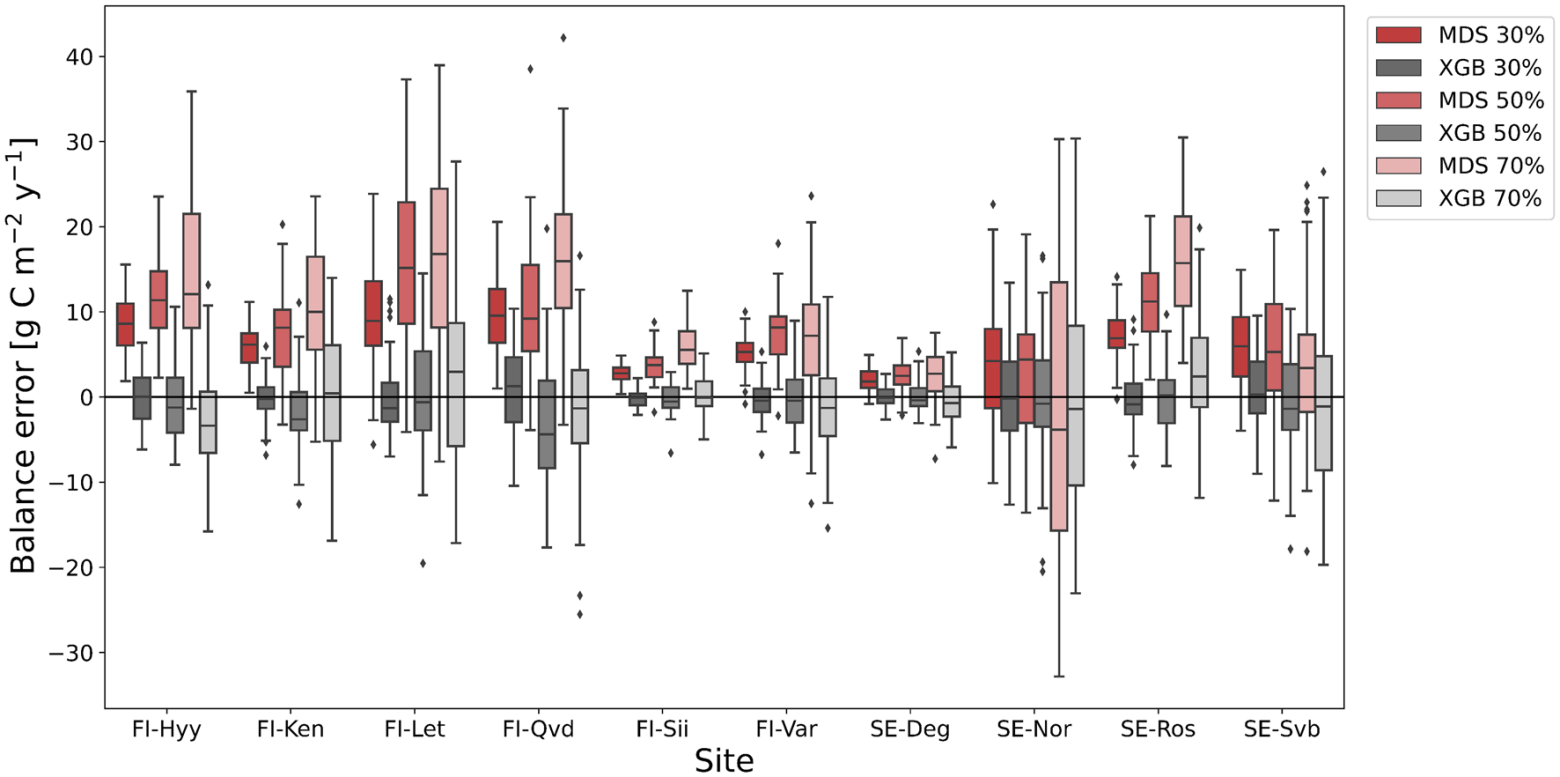
Gap-filling with MDS results in C balance errors. Errors of the gap-filled annual C balance for different gap percentages. A synthetic data set was generated five times for each site (Table 1), and each data coverage class was tested 10 times for each data set. Random gaps of 0.5 h to 3 days in length were generated based on the original gap distribution taking also into account the timing of gaps. Positive balance errors mean that the gap-filled C balance estimate indicates too large a source or too small a sink.

We found that MDS systematically overestimated the annual C balance in all except one case (site SE-Nor with 70% gaps) (Fig. 3 and Supplementary Table S3). The site-specific median balance error with MDS ranged from 2–10 g C m$^{-2}$ y$^{-1}$ at the 30% gap level to 3–17 g C m$^{-2}$ y$^{-1}$ at the 70% gap level. For 5 out of the 10 data sets, balance errors exceeded 30 g C m$^{-2}$ y$^{-1}$ and the largest balance error was 42 g C m$^{-2}$ y$^{-1}$. Noteworthy is that the annual absolute balance error was similar for sites with synthetic balances ranging from tens (FI-Qvd, FI-Let) to hundreds (FI-Hyy, SE-Ros) of grams of C m$^{-2}$ y$^{-1}$. Also, when more gaps fell into the nighttime than daytime data, the annual error of MDS decreased, because the larger number of underestimated NEE values during nighttime compensated for the smaller number of overestimated NEE during daytime. In four out of thirty cases, there was a significant error in the balance calculated with XGBoost, but the median balance errors were only − 4 to 2 g C m$^{-2}$ y$^{-1}$.

### C balance errors can be decreased by using a modified version of MDS

To test if MDS could be improved by modifying the method and to further verify that the positive flux bias was caused by the biased radiation sampling, we investigated the model performance with three alterations using the synthetic data set FI-Let, for which MDS overestimated the most. We either (1) narrowed the two tolerance settings defining the SWR limits of each data sample, (2) used only one radiation tolerance, or (3) for daytime data, calculated separately the mean NEE of low- and high-SWR subsamples and then averaged these means.

**Figure 4:**
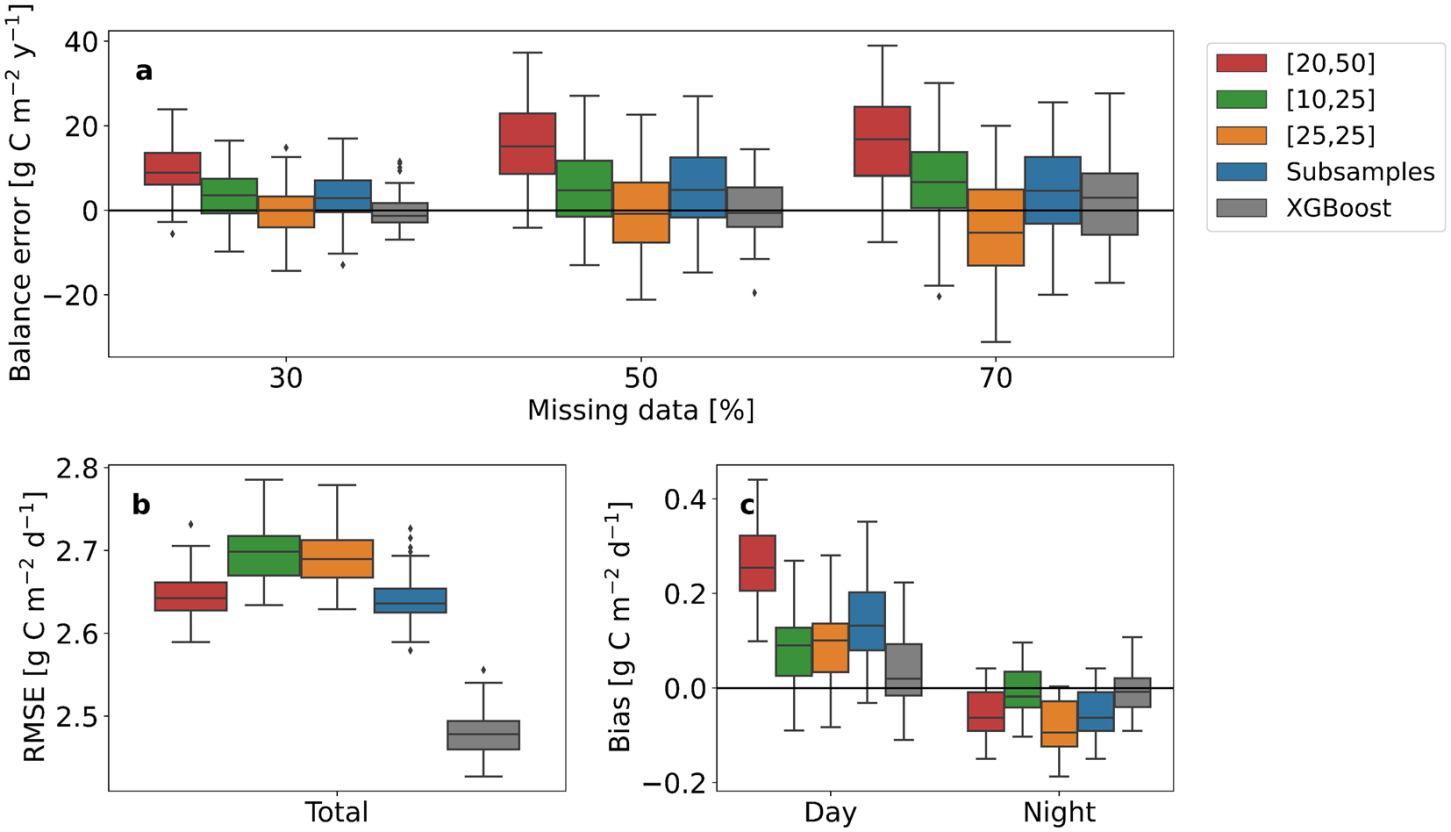
A modified version of MDS can be used to decrease C balance errors. Performance of different implementations of the MDS gap-filling method and XGBoost for the synthetic data set FI-Let. [20,50], [10,25] and [25,25] (W m$^{-2}$) indicate the sampling tolerances used for SWR. ‘Subsamples’ refers to averaging of the mean NEEs of the low- and high-SWR subsamples of data during daytime. [20,50] is the standard implementation of MDS. (**a**) Annual error, (**b**) root mean squared error (RMSE) and **c** daytime and nighttime bias of gap-filled synthetic CO$_2$ flux data. For evaluating errors, each data coverage class was tested 10 times, and RMSE and biases were calculated for data with 70% artificial gaps.

When the SWR tolerances were different for lower and higher SWR levels, the positive flux bias during daytime was larger than the negative nighttime bias, causing a positive total bias and thus an overestimation of the C balance (Fig. 4a,c and Supplementary Tables S4 and S5). When the SWR classes were narrowed, the errors were smaller compared to the original implementation, but the root mean squared error (RMSE) of modelled NEE increased (Fig. 4b and Supplementary Table S6). When a common SWR tolerance of 25 W m$^{-2}$ was used, the error of the gap-filled C balance was insignificant when 30% or 50% of data was missing and negative when 70% of data was missing (Fig. 4a and Supplementary Table S4). Also in this case RMSE increased (Fig. 4c and Supplementary Table S6). Averaging the low- and high-SWR subsamples separately and taking their mean decreased the positive daytime flux bias and the error of the annual balance without affecting the RMSE (Fig. 4a–c and Supplementary Tables 4, 5). However, the balance error was not eliminated completely and an even lower RMSE was obtained by using XGBoost. Finally, we gap-filled all the synthetic data sets using the implementation of MDS with subsample averaging and found that only in one case (SE-Nor with 70% missing data) the magnitude of the annual error was larger than when using the original implementation of MDS (Supplementary Fig. S5 and Supplementary Table S7). In all other cases the error was either decreased or eliminated completely.

## Discussion

Artificial differences among site-specific C balances can be generated by gap-filling, especially if methods with a tendency for both a negative and positive bias are used for different sites. This is a reason why flux networks have standardized their data processing. However, even when gap-filling with the same method, data imputation can lead to significant differences among sites because the biases are site-specific and dependent on data coverage. Previously, the random uncertainties at an annual timescale have been estimated to be 10–40 g C m$^{-2}$ y$^{-1}$^16,17^, uncertainties related to gap-filling 10–30 g C m$^{-2}$ y$^{-1}$^8,16^ and the total uncertainty of the C balance at nearly ideal sites less than ± 50 g C m$^{-2}$ y$^{-1}$^4^. The magnitude of the annual errors found in this study is comparable to the estimated uncertainties, meaning that in some cases the true balance might not be captured by the confidence interval of the original estimate. We showed that a machine learning algorithm, here the extreme gradient boosting technique, can substantially reduce the gap-filling error.

In principle, any method that correctly accounts for the covariation between NEE and its meteorological drivers, such as other machine learning methods and regression models, could reduce the gap-filling error. The key problem with MDS in its original implementation is that the mean flux within a certain radiation interval is a biased estimate due to the favouring of lower radiation levels and therefore higher NEE. A different but similar problem could be observed in regression models if the assumed shape of the response of NEE to radiation is incorrect. The advantage of data-driven machine learning methods is that they do not make any, or make fewer, assumptions on the response function.

Even though standard methods aid in making comparisons, there is still a need to improve these methods, in particular considering specific environmental conditions like those at northern sites. It should also be noted that the amount of gap-filled, i.e. modelled, data is typically at least 50% and, therefore, it is crucial that the modelled data are unbiased. We showed that the original implementation of MDS is a sub-optimal method for gap-filling data from northern sites where the radiation distribution is very skewed. Other methods, such as XGBoost or the proposed modified MDS, should be considered in the standardized processing pipeline of FLUXNET, at least for the northern high-latitude (latitude $>60^\circ$) sites. The results could hold also for other sites, such as southern high-latitude sites, and if applied to different drivers.

While the absolute errors found in this work may not seem large, systematic errors should be eliminated where possible. With increasing interest in the C sequestration potential of ecosystems, it is noteworthy that C balances of high-latitude ecosystems are generally small. The observed mean annual NEE has been reported to be − 17 g C m$^{-2}$ in the high-latitude boreal and tundra biomes ($>45^\circ$) which cover an area of $20.6 \times$ 10$^6$ km$^2$^18^. Therefore, the systematic gap-filling errors discovered here can have a significant relative impact on the C balance estimates of northern ecosystems, with implications for the verification of C sequestration.

## Methods

### Data

The FLUXNET2015 data set and the data acquired from an ICOS data product (Warm Winter 2020 ecosystem eddy covariance flux product for 73 stations in FLUXNET) consist of open-access eddy covariance CO$_2$ flux data and supporting measurements that have been processed in a standardized manner^6,15^. The FLUXNET data have been collected from 206 sites distributed around the globe. From the FLUXNET2015 data set, we used all site-years from the northern hemisphere that had at least 20% annual coverage (Supplementary Table S8). We utilized measured NEE by selecting those values in the data product NEE_VUT_REF where the quality flag NEE_VUT_REF_QC was zero. The selected environmental drivers were shortwave radiation (SW_IN_F), atmospheric vapour pressure deficit (VPD_F_MDS) and air temperature (TA_F_MDS). Gaps in the drivers were filled according to the FLUXNET data processing protocol^6^. From the ICOS data product we used data from all northern ($>60^\circ$) sites that had at least 30% annual coverage and T$_{air}$, VPD, SWR and soil temperature available. For each site we selected the site-year that had the highest data coverage (Table 1).

**Table 1:** Site information for ten northern sites adopted for detailed gap-filling analysis.

Site id	Year	Name	Country	Latitude	Longitude	Site type	Data coverage
FI-Hyy	2015	Hyytiala	Finland	61.84741	24.29477	Evergreen needleleaf forests	0.53
FI-Ken	2020	Kenttarova	Finland	67.98721	24.24301	Evergreen needleleaf forests	0.30
FI-Let	2012	Lettosuo	Finland	60.64183	23.95952	Evergreen needleleaf forests	0.50
FI-Sii	2020	Siikaneva	Finland	61.83265	24.19285	Permanent wetlands	0.55
FI-Var	2018	Varrio	Finland	67.7549	29.61	Evergreen needleleaf forests	0.46
FI-Qvd	2020	Qvidja	Finland	60.29550	22.39281	Agricultural grassland	0.40
SE-Deg	2006	Degero	Sweden	64.18203	19.55654	Permanent wetlands	0.63
SE-Nor	2020	Norunda	Sweden	60.0865	17.479504	Evergreen needleleaf forests	0.53
SE-Svb	2020	Svartberget	Sweden	64.25611	19.7745	Evergreen needleleaf forests	0.40
SE-Ros	2018	Rosinedal-3	Sweden	64.1725	19.738	Evergreen needleleaf forests	0.39

### Gap-filling methods

Two gap-filling methods were used in this work: extreme gradient boosting and marginal distribution sampling.

The extreme gradient boosting algorithm is based on parallel boosted decision trees. The ‘xgboost’ Python package^19^ was used to apply this method. Hyperparameters, which control for the subsample ratio of columns when constructing each tree (0.4, 0.6, 0.8, 1), the maximum depth of a tree (3, 5, 10, 15), minimum number of samples required to create a new node in a tree (2, 5, 10) and the fraction of observations that is randomly sampled for each tree (0.65, 0.75, 1) were determined for the FLUXNET2015 data based on grid search using ten randomly selected data sets and by selecting the mode of each hyperparameter. For the northern sites, the same hyperparameters were optimized for each synthetic data set using the original artificial gap-free data and grid search. For learning rate we used the default value of 0.1. The squared error was used as the loss function. Since MDS uses a moving data window to resolve temporal correlations in the flux data, XGBoost was augmented by two cyclical functions for both month and time of day, and a linear description of time as additional drivers:

1 $$\begin{aligned} t_1= & {} \,sin \left ((month-1)\times \frac{2\pi }{12} \right) \end{aligned}$$

2 $$\begin{aligned} t_2= & {} \,cos \left ((month-1)\times \frac{2\pi }{12} \right) \end{aligned}$$

3 $$\begin{aligned} t_3= & {} \,sin \left(hour\times \frac{2\pi }{24} \right) \end{aligned}$$

4 $$\begin{aligned} t_4= & {} \,cos \left(hour\times \frac{2\pi }{24}\right) \end{aligned}$$

5 $$\begin{aligned} t_5= & {} \,i \end{aligned}$$

where *i* is the number of half hours from the beginning of the year.

In MDS, gaps are filled with the average of fluxes measured under similar meteorological conditions using a moving window for data sampling, or with the mean diurnal course (MDC) if a sufficiently large sample could not be found. The default meteorological drivers and their tolerances, or accepted deviations from actual conditions, are SWR, with a tolerance of 20 W m$^{-2}$ for SWR $\le$ 50 W m$^{-2}$ and 50 W m$^{-2}$ for SWR > 50 W m$^{-2}$, T$_{air}$ with a tolerance of 2.5 K and VPD with a tolerance of 5 hPa. If T$_{air}$ or VPD is missing, only SWR is used. If none of the meteorological drivers are available, the gaps are filled with MDC. The specific sampling procedure is described in Wutzler et al.^12^. Noteworthy is that also the REddyProc tool uses SWR tolerances of 20 W m$^{-2}$ and 50 W m$^{-2}$ (https://github.com/bgctw/REddyProc/tree/1.1.3) even though a single tolerance approach is reported.

In addition to the standard implementation of MDS, we tested modified versions of it. First, we only altered the radiation sampling limits. We tested using two different tolerances as in the original implementation, but lowered the SWR tolerances to 10 and 25 W m$^{-2}$. We also tested using only one tolerance at all radiation levels and used an SWR tolerance of 25 W m$^{-2}$ for this. Finally, to better account for the sample bias, for daytime data, we first calculated the average NEE separately for the subsamples of data with higher and lower SWR than the present SWR and then averaged these two NEE values. The MDS variants were implemented using a C code by Papale et al.^20^.

### Synthetic data

An artificial neural network (ANN) was used to generate synthetic data sets with 100% coverage that could be used for comparing the gap-filling methods. The ANN used here was a sequential model with four hidden layers, with 16 nodes in the first hidden layer and 32 in the other layers. The activation functions that were used were linear, hyperbolic tangent (tanh) and rectified linear activation (relu), and the structure of the network was linear-tanh-tanh-relu-linear. Mean squared error was used as the loss function. The ANN was implemented using the Keras library^21^. Air temperature, soil temperature, SWR and VPD were used as predictors for the neural network. For each site, we used all the available measured data to train the ANN and after modelling for all 30-min periods of one year, noise was added to the modelled 30-min NEE. This was done by binning the model residuals based on the season (winter months 1–4 and 11–12, and summer months 5–10), time of day (nighttime with SWR $\le$ 20 W m$^{-2}$ and daytime with SWR > 20 W m$^{-2}$ ), and air temperature (five bins of equal size). After binning the data, a residual was randomly selected from the correct bin and added to each 30-min NEE. The whole procedure was repeated five times for each site to obtain 50 different synthetic data sets.

In practice, the synthetic data represent a case in which NEE is driven by T$_{air}$, SWR, VPD and soil temperature. We assumed that there is noise in the data and that it is of different magnitude during the active summer months and winter, during daytime and night and in different temperatures. When gap-filling the data with MDS and XGBoost, we assumed that they should be able to solve the covariation between NEE and T$_{air}$, SWR and VPD. We also assumed that the average errors of an unbiased gap-filling method would be zero.

### Artificial gaps

Of the FLUXNET2015 data, we sampled all available measured data to calculate the mean bias for each site-year. For MDS we labelled one half hour at a time as an artificial gap since it is easy to implement in a moving window. For XGBoost we labelled one percent of the measured data as artificial gaps at a time since training a different model for each half hour was not reasonable due to computational time constraints. However, the results for XGBoost represent a conservative estimate since training a separate model for each individual half hour would improve rather than worsen the results.

For the synthetic data sets, the artificial gaps were drawn from the original gap distributions, taking into account both gap length and timing. More specifically, the gaps in the original time series were labelled by their length and starting time, and the artificial gaps were randomly chosen from the original gaps and inserted into the synthetic data sets until 30%, 50% or 70% coverage was achieved. When inserting the artificial gaps into the synthetic data, the starting time of each gap was retained. The artificial gaps were not allowed to overlap. For each data coverage we generated ten different gap sequences that were used for all synthetic data sets.

### Performance measures

Comparisons between gap-filled and measured values were carried out using mean bias, RMSE and the error of the annual gap-filled C balance. In this paper, we defined the C balance as the C balance measured with EC excluding the potential impact of harvests, fertilization, leaching and lateral transport of C.

Mean bias and RMSE were defined as:

6 $$\begin{aligned} \textit{Bias}= \frac{1}{N} \sum _{n=1} ^{N}{(NEE_{gap,i}-NEE_{meas,i})} \end{aligned}$$

7 $$\begin{aligned} \textit{RMSE}= \sqrt{ \frac{\sum _{n=1} ^{N}{(NEE_{gap,i}-NEE_{meas,i})^2}}{N} } \end{aligned}$$

where N equals the amount of artificial gaps, and NEE$_{gap, i}$ indicates a gap-filled and NEE$_{meas, i}$ a measured value.

The error of the gap-filled C balance was calculated as the difference between the gap-filled and true (synthetic) C balances:

8 $$\begin{aligned} \textit{Balance error} = \sum _{n=1} ^{N}{NEE_{gap, i}}-\sum _{n=1} ^{N}{NEE_{synth, i}} \end{aligned}$$

where N equals the number of 30-min NEE in the data and NEE$_{synth, i}$ a synthetic NEE value.

### Statistical tests

To determine whether the mean biases of half hourly NEE and the errors of the gap-filled carbon balances differed from zero, we used the non-parametric Wilcoxon signed-rank test. Normality of the data was first evaluated using the Shapiro–Wilk test. Pairwise comparisons were done using Conover’s test and Holm’s method to adjust p-values. All statistical tests were performed in Python using the SciPy package^22^.

## Supplementary Information

Supplementary Information 1.

Supplementary Legends.

Supplementary Table S8.

## Data Availability

The FLUXNET2015 data set is available at http://fluxnet.fluxdata.org/data/ fluxnet2015-dataset/. The ICOS Warm Winter 2020 ecosystem eddy covariance flux product for 73 stations in FLUXNET-Archive format-release 2022-1 is available at http://www.icos-cp.eu/data-products/2G60-ZHAK.

## Code availability

Code for making synthetic data and gap-filling using XGBoost is available at https://github.com/hvekuri/co2_gapfilling.
