# Supplementary material for: A widely-used eddy covariance gap-filling method creates systematic bias in carbon balance estimates
Source: Sci Rep. 2023 Jan 31;13:1720. doi: 10.1038/s41598-023-28827-2 (PMC9889393; doi:10.1038/s41598-023-28827-2)
Supplement: Supplementary file 1 — Supplementary Information 1. [file 41598_2023_28827_MOESM1_ESM.pdf]

Supplementary information for

A widely-used eddy covariance gap-filling method creates systematic bias in carbon balance estimates

Henriikka Vekuri*[1], Juha-Pekka Tuovinen[1], Liisa Kulmala[1], Dario Papale[2,3], Pasi Kolari[4], Mika Aurela[1], Tuomas Laurila[1], Jari Liski[1], Annalea Lohila [1,4]

[1]Finnish Meteorological Institute, Helsinki, 00101, Finland

[2]DIBAF University of Tuscia, Viterbo, 01100, Italy

[3]Euro-Mediterranean Center on Climate Change CMCC IAFES, Viterbo, 01100, Italy

[4]Institute for Atmospheric and Earth System Research, Physics, University of Helsinki, Helsinki, 00014, Finland

*henriikka.vekuri@fmi.fi

*

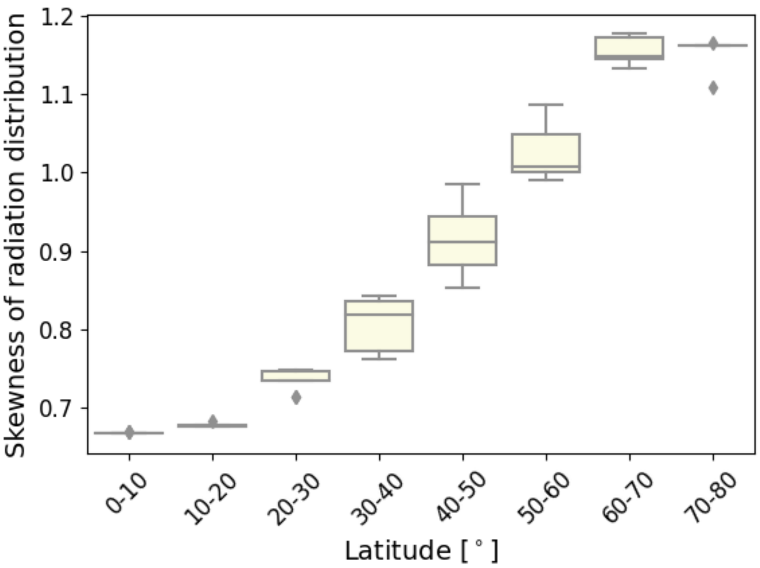

Figure 1: Skewness of the distribution of potential shortwave incoming radiation (top of atmosphere theoretical maximum radiation calculated based on site coordinates) at different latitudes.

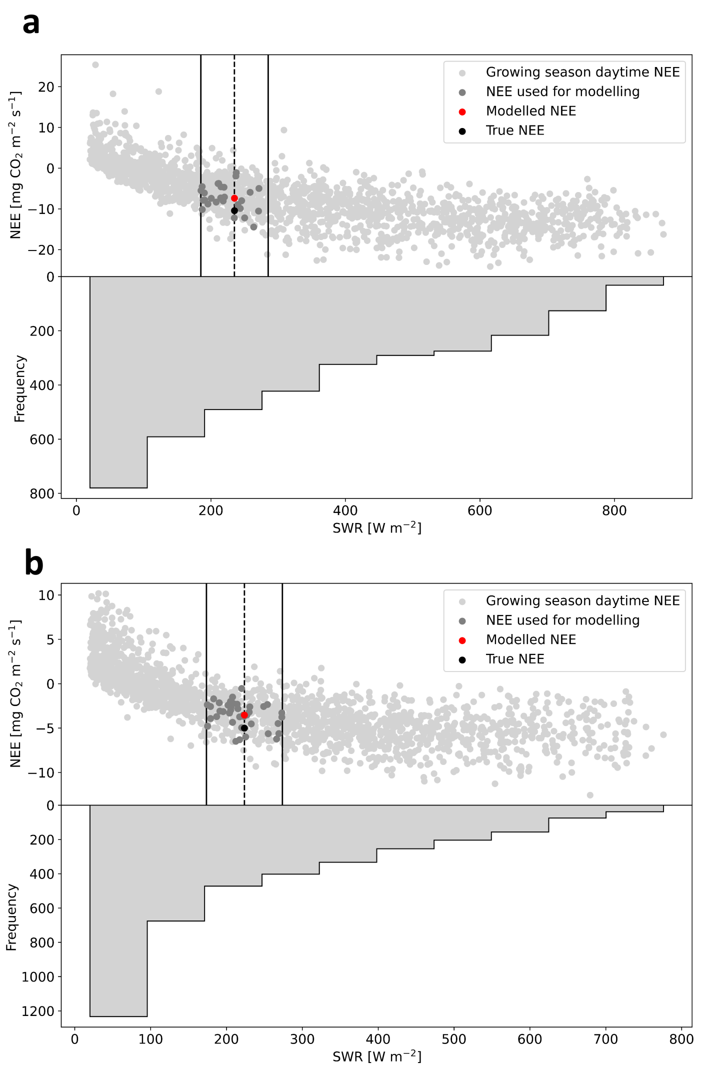

Figure 2: Examples how the positive bias is produced when using MDS for gap-filling. The data is from daytime and growing season (months 6-8) The dashed vertical line shows measured SWR and the two other vertical lines the accepted interval for NEE that is used for modelling. The lower panels show the frequency of available data in different radiation levels. (**a**) is from the site from FI-Let (2010) and (**b**) from FI-Sod (2001).

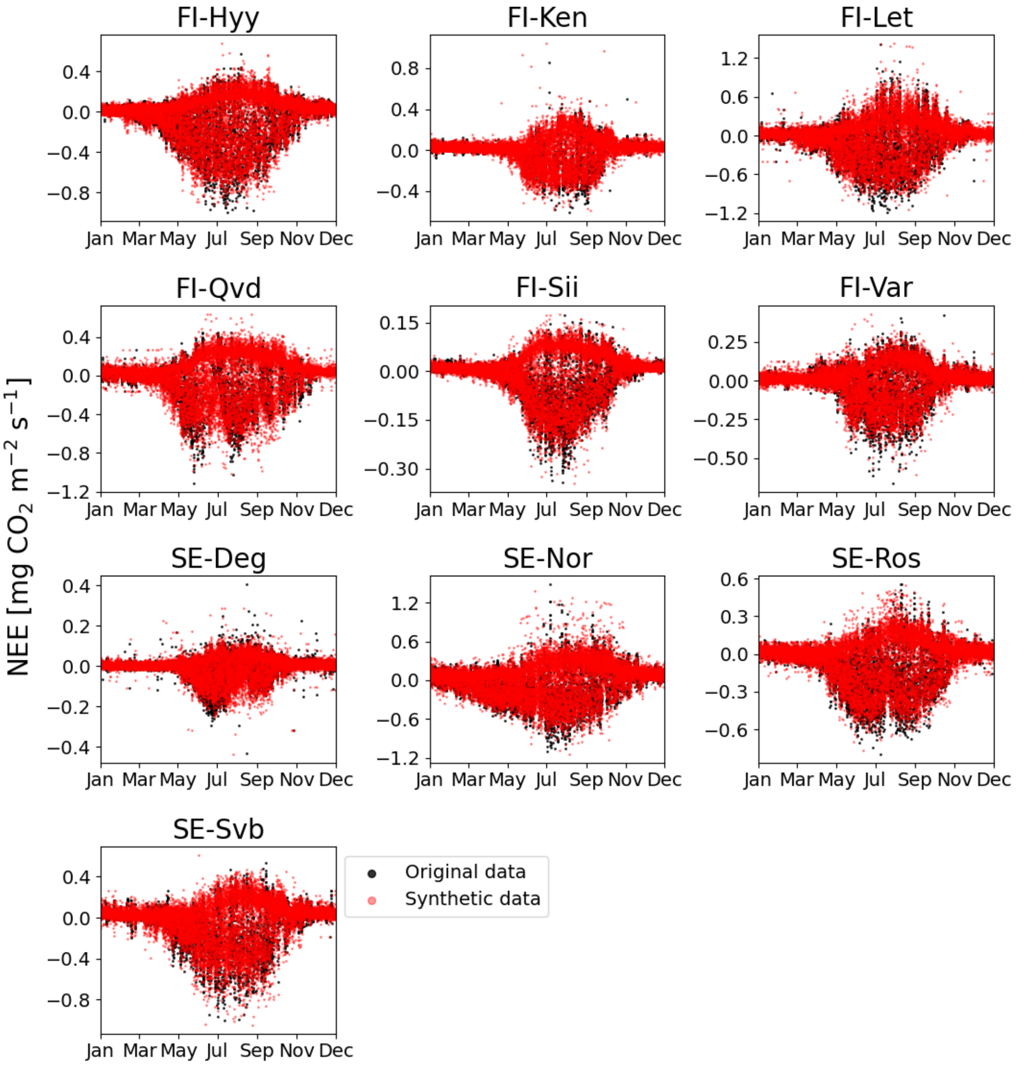

Figure 3: Examples of time series of synthetic NEE data and the corresponding original measured data for each northern site.

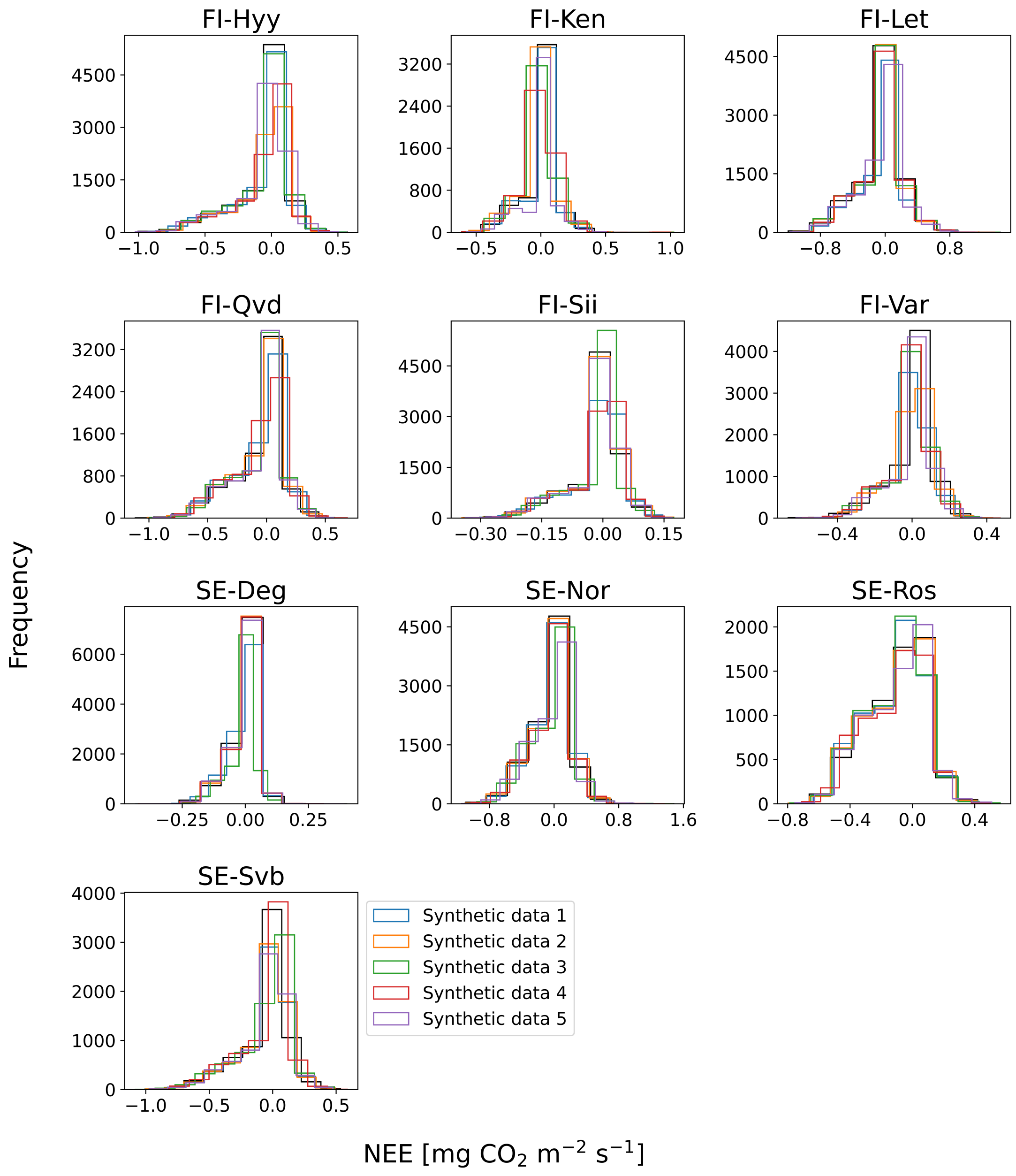

Figure 4: Distributions of original and synthetic NEE data. Of the synthetic data sets, only the values with corresponding measured data are plotted for comparability. Black histograms indicate the original measured data and coloured histograms the synthetic data.

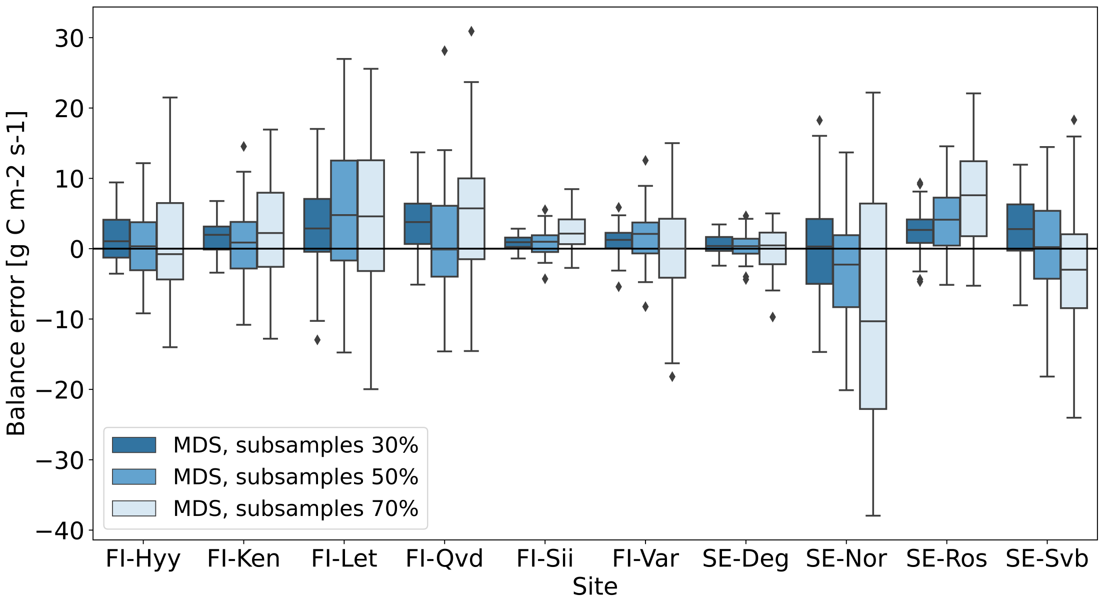

Figure 5: Errors of the gap-filled annual C balance for different gap percentages and synthetic data for different sites. Subsamples refers to first calculating the mean of high- and low-SWR subsamples of data and then taking their mean for daytime data.

Table 1: Median bias and number of site-years with a positive bias (bias > 1 mg C m^{-2} d^{-1} and p-value < 0.01) and negative bias (bias < -1 mg C m^{-2} d^{-1} and p-value < 0.01) for all, daytime and nighttime CO_2 flux (mg C m^{-2} d^{-1}) for different latitude bands (°) in FLUXNET2015 data. Significance was evaluated using Wilcoxon signed rank tests.

			MDS			XGBoost		
Time	Latitude	N	Median flux bias	N positive bias	N negative bias	Median flux bias	N positive bias	N negative bias
Total	0-10	21	15	0	0	3	0	0
Total	10-20	4	0	1	0	1	0	0
Total	20-30	9	25	0	0	0	0	0
Total	30-40	133	-2	14	11	0	5	2
Total	40-50	427	-4	57	45	1	16	7
Total	50-60	242	17	99	8	-2	1	3
Total	60-70	40	46	34	0	0	0	0
Total	70-80	6	22	0	0	1	0	0
Day	0-10	21	5	1	0	4	0	0
Day	10-20	4	2	0	0	0	0	0
Day	20-30	9	41	0	0	1	0	0
Day	30-40	133	-3	6	8	1	1	4
Day	40-50	427	-6	30	27	2	9	2
Day	50-60	242	38	71	0	1	0	0
Day	60-70	40	93	33	0	1	0	0
Day	70-80	6	40	1	0	2	0	0
Night	0-10	21	-6	1	0	-13	0	0
Night	10-20	4	-4	0	1	1	0	0
Night	20-30	9	-7	1	1	-3	0	1
Night	30-40	133	-1	17	29	-2	6	8
Night	40-50	427	-4	54	79	-3	15	65
Night	50-60	242	-10	15	56	-4	6	46
Night	60-70	40	-12	0	2	-2	0	3
Night	70-80	6	-15	0	3	0	0	0

Table 2: Annual C balance (g C m^{-2}), mean, median and standard deviation (SD) of fluxes (mg CO_2 m^{-2} s^{-1}) and other descriptive statistics of real and synthetic data sets.

Site	Type	Annual C balance	Mean flux	Median flux	SD of flux	Skewness	Kurtosis
FI-Hyy	Real		-0.062	0.0130	0.20	-1.48	2.16
FI-Hyy	Synthetic	-319	-0.062	0.013	0.20	-1.36	1.60
FI-Hyy	Synthetic	-312	-0.061	0.012	0.19	-1.31	1.52
FI-Hyy	Synthetic	-310	-0.061	0.013	0.20	-1.39	1.68
FI-Hyy	Synthetic	-318	-0.063	0.013	0.20	-1.35	1.51
FI-Hyy	Synthetic	-312	-0.062	0.014	0.20	-1.40	1.71
FI-Ken	Real		-0.008	0.020	0.13	-0.84	2.33
FI-Ken	Synthetic	97	-0.007	0.020	0.13	-0.66	1.53
FI-Ken	Synthetic	97	-0.009	0.021	0.13	-0.68	2.10
FI-Ken	Synthetic	87	-0.009	0.020	0.13	-0.67	2.41
FI-Ken	Synthetic	102	-0.009	0.021	0.13	-0.71	1.54
FI-Ken	Synthetic	93	-0.009	0.022	0.13	-0.76	1.95
FI-Let	Real		-0.053	0.018	0.28	-0.64	1.31
FI-Let	Synthetic	18	-0.053	0.017	0.28	-0.56	0.90
FI-Let	Synthetic	33	-0.053	0.017	0.28	-0.56	0.94
FI-Let	Synthetic	32	-0.052	0.018	0.28	-0.58	0.80
FI-Let	Synthetic	27	-0.052	0.018	0.28	-0.55	1.09
FI-Let	Synthetic	29	-0.052	0.018	0.27	-0.56	0.99
FI-Qvd	Real		-0.061	0.028	0.21	-1.10	1.01
FI-Qvd	Synthetic	-42	-0.063	0.029	0.22	-0.95	0.52
FI-Qvd	Synthetic	-51	-0.061	0.023	0.21	-0.96	0.66
FI-Qvd	Synthetic	-35	-0.062	0.025	0.22	-1.02	0.70
FI-Qvd	Synthetic	-36	-0.060	0.029	0.22	-0.96	0.52
FI-Qvd	Synthetic	-29	-0.061	0.023	0.22	-0.95	0.66
FI-Sii	Real		-0.016	0.007	0.06	-1.39	2.09
FI-Sii	Synthetic	-19	-0.016	0.007	0.07	-1.28	1.48
FI-Sii	Synthetic	-22	-0.016	0.007	0.07	-1.27	1.33
FI-Sii	Synthetic	-24	-0.016	0.007	0.06	-1.25	1.36
FI-Sii	Synthetic	-18	-0.015	0.008	0.07	-1.25	1.45
FI-Sii	Synthetic	-22	-0.016	0.007	0.07	-1.24	1.38
FI-Var	Real		-0.009	0.015	0.12	-1.13	2.13
FI-Var	Synthetic	3	-0.010	0.014	0.12	-0.96	1.54
FI-Var	Synthetic	11	-0.009	0.015	0.11	-0.88	1.02
FI-Var	Synthetic	4	-0.010	0.015	0.12	-1.00	1.27
FI-Var	Synthetic	5	-0.011	0.014	0.12	-1.00	1.32
FI-Var	Synthetic	3	-0.010	0.015	0.12	-0.99	1.29
SE-Deg	Real		-0.010	0.003	0.05	-1.23	3.59
SE-Deg	Synthetic	-22	-0.010	0.003	0.05	-1.09	2.97
SE-Deg	Synthetic	-19	-0.010	0.003	0.05	-0.90	2.99
SE-Deg	Synthetic	-24	-0.010	0.003	0.05	-0.93	2.65
SE-Deg	Synthetic	-22	-0.010	0.003	0.05	-0.99	2.84
SE-Deg	Synthetic	-22	-0.010	0.003	0.06	-0.88	2.67
SE-Nor	Real		-0.033	0.045	0.25	-0.48	1.31
SE-Nor	Synthetic	212	-0.035	0.041	0.24	-0.46	0.61
SE-Nor	Synthetic	221	-0.032	0.040	0.25	-0.42	0.63
SE-Nor	Synthetic	227	-0.033	0.043	0.25	-0.36	1.10
SE-Nor	Synthetic	208	-0.033	0.038	0.25	-0.34	0.90
SE-Nor	Synthetic	239	-0.033	0.042	0.25	-0.46	0.51
SE-Ros	Real		-0.108	-0.057	0.19	-0.43	-0.29
SE-Ros	Synthetic	-261	-0.106	-0.055	0.19	-0.37	-0.52
SE-Ros	Synthetic	-267	-0.108	-0.055	0.19	-0.38	-0.51
SE-Ros	Synthetic	-271	-0.108	-0.056	0.19	-0.37	-0.51
SE-Ros	Synthetic	-254	-0.106	-0.051	0.19	-0.40	-0.49
SE-Ros	Synthetic	-261	-0.107	-0.055	0.19	-0.38	-0.56
SE-Svb	Real		-0.057	0.016	0.20	-1.27	1.68
SE-Svb	Synthetic	-143	-0.056	0.016	0.20	-1.23	1.60
SE-Svb	Synthetic	-156	-0.057	0.015	0.20	-1.19	1.60
SE-Svb	Synthetic	-143	-0.056	0.015	0.20	-1.09	1.20
SE-Svb	Synthetic	-138	-0.056	0.017	0.20	-1.20	1.39
SE-Svb	Synthetic	-148	-0.055	0.016	0.20	-1.20	1.55

Table 3: Significance of annual errors (g C m^{-2} y^{-1}) of the gap-filled C balances for synthetic data sets with different amounts of missing data (%), evaluated using Wilcoxon signed rank tests. $p<0.05$ are in bold. N = 50 for each site and gap percentage combination.

		MDS		XGBoost	
Site	Gap percentage	Median balance error	p-value	Median balance error	p-value
FI-Hyy	30	9	**<0.001**	0	0.958
FI-Hyy	50	11	**<0.001**	-1	0.299
FI-Hyy	70	12	**<0.001**	-3	**0.022**
FI-Ken	30	6	**<0.001**	0	0.508
FI-Ken	50	8	**<0.001**	-3	**0.002**
FI-Ken	70	10	**<0.001**	0	0.866
FI-Let	30	9	**<0.001**	-1	0.166
FI-Let	50	15	**<0.001**	-1	0.851
FI-Let	70	17	**<0.001**	3	0.178
FI-Qvd	30	10	**<0.001**	1	0.191
FI-Qvd	50	9	**<0.001**	-4	**0.002**
FI-Qvd	70	16	**<0.001**	-1	0.124
FI-Sii	30	3	**<0.001**	0	0.197
FI-Sii	50	4	**<0.001**	-1	0.521
FI-Sii	70	6	**<0.001**	0	0.431
FI-Var	30	5	**<0.001**	0	0.277
FI-Var	50	8	**<0.001**	0	0.585
FI-Var	70	7	**<0.001**	-1	0.149
SE-Deg	30	2	**<0.001**	0	0.889
SE-Deg	50	2	**<0.001**	0	0.585
SE-Deg	70	3	**<0.001**	-1	0.146
SE-Nor	30	4	**0.001**	0	0.935
SE-Nor	50	4	**0.013**	-1	0.973
SE-Nor	70	-4	0.265	-1	0.640
SE-Ros	30	7	**<0.001**	-1	0.521
SE-Ros	50	11	**<0.001**	0	0.675
SE-Ros	70	16	**<0.001**	2	**0.014**
SE-Svb	30	6	**<0.001**	0	0.277
SE-Svb	50	5	**<0.001**	-1	0.654
SE-Svb	70	3	**0.006**	-1	0.215

Table 4: Significance of errors (g C m^{-2}) of the gap-filled C balances using different amounts of missing data (%) for XGBoost and modified implementations of MDS evaluated using Wilcoxon signed rank tests. [20,50], [10,25] and [25,25] (W m^{-2}) refer to the tolerances used for shortwave radiation in MDS. Subsamples refers to first calculating the mean of high- and low-SWR subsamples of data and then taking their mean for daytime data. Data are from FI-Let. $p<0.05$ are in bold. N = 50 for each method and gap percentage combination.

Method	Gap percentage	Median balance error	p-value
MDS, [20,50]	30	9	**<0.001**
MDS, [20,50]	50	15	**<0.001**
MDS, [20,50]	70	17	**<0.001**
MDS, [10,25]	30	4	**<0.001**
MDS, [10,25]	50	5	**<0.001**
MDS, [10,25]	70	7	**<0.001**
MDS, [25,25]	30	0	0.647
MDS, [25,25]	50	-1	0.352
MDS, [25,25]	70	-5	**0.015**
MDS, Subsamples	30	3	**0.001**
MDS, Subsamples	50	5	**0.001**
MDS, Subsamples	70	5	**0.017**
XGBoost	30	0	0.658
XGBoost	50	-1	**0.001**
XGBoost	70	0	0.316

Table 5: Significance of daytime and nighttime bias (mg C m^{-2} d^{-1}) for XGBoost and modified implementations of MDS evaluated using Wilcoxon signed rank tests. [20,50], [10,25] and [25,25] (W m^{-2}) refer to the tolerances used for shortwave radiation. Subsamples refers to first calculating the mean of high- and low-SWR subsamples of data and then taking their mean for daytime data. Data are from FI-Let with 70% missing data. $p<0.05$ are in bold. N = 50 for each method.

Method	Time	Median flux bias	p-value
MDS, [20,50]	Night	-63	**<0.001**
MDS, [10,25]	Night	-18	0.277
MDS, [25,25]	Night	-94	**<0.001**
MDS, Subsamples	Night	-63	**<0.001**
XGBoost	Night	-8	**<0.001**
MDS, [20,50]	Day	254	**<0.001**
MDS, [10,25]	Day	90	**<0.001**
MDS, [25,25]	Day	100	**<0.001**
MDS, Subsamples	Day	132	**<0.001**
XGBoost	Day	8	**<0.001**

Table 6: Differences in RMSE with XGBoost and modified implementations of MDS evaluated with Conover's test and Holm's method to adjust p-values. [20,50], [10,25] and [25,25] (W m^{-2}) refer to tolerances used for shortwave radiation. Subsamples refers to first calculating the mean of high- and low-SWR subsamples of data and then taking their mean for daytime data. p<0.05 are in bold. N = 50 for each method.

	MDS, [10,25]	MDS, [20,50]	MDS, [25,25]	MDS, Subsamples	XGBoost
MDS, [10,25]	1.0				
MDS, [20,50]	**0.010**	1.0			
MDS, [25,25]	1.0	**0.013**	1.0		
MDS, Subsamples	**0.006**	1.0	**0.010**	1.0	
XGBoost	**<0.001**	**<0.001**	**<0.001**	**<0.001**	1.0

Table 7: Significance of annual errors (g C m^{-2} y^{-1}) of the gap-filled C balances for synthetic data sets with different amounts of missing data (%), evaluated using Wilcoxon signed rank tests. Data were gap-filled using a modified implementation of MDS which used the average of the means of high- and low-SWR subsamples of data to impute missing daytime data. p<0.05 are in bold. N = 50 for each site and gap percentage combination.

Site	Gap percentage	Median balance error	p-value
FI-Hyy	30	1	**0.004**
FI-Hyy	50	0	0.689
FI-Hyy	70	-1	0.791
FI-Ken	30	2	**<0.001**
FI-Ken	50	1	0.347
FI-Ken	70	2	**0.043**
FI-Let	30	3	**0.001**
FI-Let	50	5	**0.001**
FI-Let	70	5	**0.017**
FI-Qvd	30	4	**<0.001**
FI-Qvd	50	0	0.592
FI-Qvd	70	6	**<0.001**
FI-Sii	30	1	**<0.001**
FI-Sii	50	1	**0.002**
FI-Sii	70	2	**<0.001**
FI-Var	30	1	**0.001**
FI-Var	50	2	**0.002**
FI-Var	70	0	0.739
SE-Deg	30	0	**0.004**
SE-Deg	50	0	0.078
SE-Deg	70	0	0.791
SE-Nor	30	0	0.927
SE-Nor	50	-2	**0.005**
SE-Nor	70	-10	**0.001**
SE-Ros	30	3	**<0.001**
SE-Ros	50	4	**<0.001**
SE-Ros	70	8	**<0.001**
SE-Svb	30	3	**0.002**
SE-Svb	50	0	0.527
SE-Svb	70	-3	**0.022**
