# Supplementary material for: A widely-used eddy covariance gap-filling method creates systematic bias in carbon balance estimates
Source: Sci Rep. 2023 Jan 31;13:1720. doi: 10.1038/s41598-023-28827-2 (PMC9889393; doi:10.1038/s41598-023-28827-2)
Supplement: Supplementary file 2 — Supplementary Legends. [file 41598_2023_28827_MOESM2_ESM.docx]

Data acquired from the FLUXNET2015 data set (Northern Hemisphere, at least 20% data coverage) for this work. The sites were categorized according to their vegetation type based on the International Geosphere–Biosphere Programme (IGBP) definition. ENF = Evergreen Needleleaf Forest, EBF = Evergreen Broadleaf Forest, DNF = Deciduous Needleleaf Forest, DBF = Deciduous Broadleaf Forest, MF = Mixed Forest, CSH = Closed Shrubland, OSH = Open Shrubland, GRA = Grassland, SAV = Savanna, WSA = Woody Savanna, WET = Wetland, CRO = Cropland.
